# Future mainstream platform for online adaptive radiotherapy will be using on‐board MR rather than on‐board (CB) CT images

**DOI:** 10.1002/acm2.13352

**Published:** 2021-07-18

**Authors:** Daniel E. Hyer, Bin Cai, Yi Rong

**Affiliations:** ^1^ Radiation Oncology University of Iowa Iowa City IA USA; ^2^ Radiation Oncology University of Texas Southwestern Medical Center Dallas TX USA; ^3^ Radiation Oncology Mayo Clinic Arizona Phoenix AZ USA

## 1 INTRODUCTION

As several major non‐inferiority phase 3 clinical trial studies published their promising results,^1^, ^2^ and as the reimbursement pattern is reforming from fee‐for‐service payment to episode‐based payment,^3^ the radiation oncology field is undergoing a major paradigm shift, from a majority of standard fractionation treatments to more hypo‐fractionation and stereotactic body radiotherapy (SBRT). With this change, the need to verify daily radiotherapy dose and adapt the radiotherapy plan according to daily anatomy has become essential.^4^ Currently, the most common platforms for online adaptive radiotherapy (ART) include MRI‐guided ART (MRgART) and CT (or cone‐beam CT)‐guided ART (CTgART), both of which have only been adopted by a handful of large‐scale hospital‐based cancer centers around the world. As more experience is collected and technology matures, one would hope that advanced online ART platforms should become more affordable and accessible to a larger community. That brings this month's debate topic: “*Future mainstream platform for online adaptive radiotherapy will be using on*‐*board MR rather than on*‐*board (CB) CT images*.” Herein, we invited Dr. Daniel Hyer and Dr. Bin Cai to join us for this debate.

Parallel to this opinion is Dr. Daniel Hyer. Dr. Hyer received his PhD in Medical Physics from the University of Florida in 2010 and was certified by the American Board of Radiology in 2013. Dr. Hyer is currently an Associate Professor and the Director of Clinical Physics at the University of Iowa. His research interests include MRI‐guided radiotherapy and proton beam therapy. On the latter topic, Dr. Hyer currently holds a National Cancer Institute grant for the development of a proton collimator. Clinically, he is the technical director of the Elekta Unity program at the University of Iowa and has been involved with the project since 2017.

Opposed to this opinion is Dr. Bin Cai. Dr. Cai is Associate Professor and Director of Advanced Physics Service in the Department of Radiation Oncology at the University of Texas Southwestern Medical Center. Dr. Cai received his Ph.D. in Physics from Ohio University and completed his medical physics residency training at Washington University School of Medicine. Dr. Cai stayed as a faculty member for  6 years at Washington University after residency. In 2021, he joined UT Southwestern to help develop the adaptive radiotherapy program as well as to lead the implementation of biology‐guided radiotherapy. Dr. Cai is one of the key team members that led the clinical implementation of the world's first MRgART platform. Later he also led the clinical development and implementation of the first CBCT‐guided online adaptations in the United States. Therefore Dr. Cai has extensive and balanced experience on both CTgART and MRgART platforms.

## 2 OPENING STATEMENTS

### 2.1 Daniel E. Hyer, PhD

The primary goal of online ART is to enable clinicians to develop treatment plans that account for daily anatomical changes of both targets and surrounding normal tissues. Each specific clinical indication has its own set of challenges, and to address these varied issues, clinicians need tools. My esteemed colleague in this debate will be forced to argue that the enormous variety of clinical challenges can entirely or mostly be managed with one tool: a non‐contrast on‐board (CB) CT. I maintain that the way forward for our field is to build not on a single imaging modality, but on a toolset that can be modified to visualize essentially any tissue in the body. This toolset, represented by MR‐Linacs, are equipped with a library of imaging sequences to visualize and enhance a variety of tissues in the body. More importantly, we are currently only scratching the surface of the potential variety of MRI sequences that may have clinical utility in the field of radiotherapy. The non‐contrast on‐board (CB) CTs have and will continue to play an important role in radiotherapy, but the future of online adaptive radiotherapy is the MR toolset.

To inform us on what the future of adaptive radiotherapy holds, I believe it is important to consider the historic use of CT in radiotherapy. CT was adopted to provide clinicians with a 3D image to visualize targets and organs at risk that has (1) accurate spatial integrity and (2) density information necessary for dose calculations. If we consider these two features in the context of modern MR scanners, we find that the historical challenges to using MR for these two goals have been addressed: spatial distortion concerns have been managed ^5^ and density requirements can be addressed by the generation of a synthetic CT.^6^ MRI‐only simulation has already been demonstrated feasible for brain treatments^7^ and trends in machine learning show promise for other anatomical sites as well.^8^ In addition, image registration uncertainties for combining MRI with CT in the traditional treatment planning paradigm can be eliminated with the use of an MRI‐only simulation. This paradigm shift toward MRI‐only simulation can and will be propagated to daily adaptive radiotherapy—bringing with it the potential to provide equivalent or better information than on‐board (CB) CT‐based adaptation while sparing unnecessary and non‐specific imaging dose daily.

The soft‐tissue contrast afforded by MRI has proven critical in numerous clinical cases in our department, but I would like to highlight one especially exciting trial which suggests an increase in 2‐year overall survival for inoperable pancreatic cancer with the use of an MR‐Linac.^9^ In this trial, the ability to visualize the surrounding organs at risk (duodenum, stomach, bowel) was used for iso‐toxic planning, whereby the dose to the target tissue was escalated on days with favorable anatomy. It was observed that patients who received greater radiation dose (BED_10_ > 70 Gy) had significant improvements in 2‐year overall survival compared with patients who received a standard treatment regimen in the same trial. Pancreatic dose escalation to this level is likely not possible without the use of MRI‐guided adaptive therapy to delineate the surrounding organs at risk and manage motion in real‐time.

From a technical standpoint, on‐board MRI not only provides high‐quality 3D images, but also enables continuous 2D cine imaging of the patient during treatment with no extraneous radiation dose. MR‐Cine during daily treatment allows for respiratory gating based on nearly real‐time tumor images rather than the use of a surrogate such as implanted fiducials, as is often required for x‐ray‐guided radiotherapy. This is a notable breakthrough for the treatment of moving targets and has the potential to reduce margins as the uncertainties associated with respiratory gating are greatly mitigated when the user has the ability to track the actual tumor while also visualizing adjacent organs at risk. In the future, it is expected that MRI‐based treatment systems will advance beyond gating for moving targets, and toward MLC tracking based on these cine images—this is something that x‐ray imaging will only be able to do with fiducial implants for targets outside of the lung.

The final area where MRI holds an advantage over (CB) CT‐based online adaptive therapy is in the growing field of quantitative imaging. There is still much work to do in this area, however, promising data are emerging for tumor response evaluation using MR sequences such as diffusion‐weighted imaging,^10^ as well as sub‐volume dose escalation within target tissues based on hypoxia sequencing.^11^ While it is too early to determine the role that quantitative imaging will play in the future of radiation therapy, these promising initial results will only be possible with an MRI radiotherapy platform; (CB) CT platforms simply lack the requisite versatility to capture the underlying biology.

I can comfortably concede that non‐contrast on‐board (CB) CT will continue to be the mainstay with respect to image‐guided radiation therapy—not all situations warrant adaptive therapy. However, for the more technically and clinically challenging scenario of online adaptive therapy, we will need and want the full panoply of tools that MR imaging brings.

### 2.2 Bin Cai, Ph.D.

With on‐board MR or X‐ray imaging system, both MR‐guided and CT‐guided online adaptive radiotherapy (MRgART and CTgART) have been implemented clinically by early adopters. Such plan adaption compensates for daily anatomy changes or set up uncertainties, therefore, enables target dose hypofractionation and/or escalation while maintaining low toxicities to organs at risk (OARs). The first commercial MRgART platform, using Co‐60 sources, was deployed to the clinic back in 2014,^12^ and current state‐of‐the‐art technology provided by several vendors couples Linac and MR with various magnetic field strengths.^5^, ^13^ More recently, the integrated CTgART commercial solutions were implemented utilizing either on‐board kV Fan‐beam (FB) (ClearRT™, Accuray Inc. Sunnyvale, CA; uRT‐linac 506c, United Imaging Healthcare Co., Ltd, Shanghai, China) or Cone‐beam (CB) CT (Ethos™, Varian Medical Systems, Palo Alto, CA). Both MRgART and CTgART have shown promising results in improving dosimetry accounting for daily anatomy changes, and many clinical trials are underway looking into clinical outcome benefits for a variety of disease sites. In comparison with CTs, on‐board MR images offer superior soft‐tissue contrast which is especially beneficial to abdominal areas. However, the clinical implementation of MRgART poses more challenges compared to CTgART in various aspects. The main challenges are multi‐fold: 1. high initial cost for vault construction, treatment machines, MR compatible accessories/tools, and MR‐trained personnel; 2. clinical workflow with high complexities and uncertainties, requiring dedicated personnel time and extra effort; and 3. low patient throughput and potential MR hazard further restricting MRgART adoption by small radiotherapy centers.

First, the implementation of MRgART requires significant initial investment in treatment vault construction, MRgART machine with custom‐designed hardware accessories or tools, as well as extra staffing with MR imaging experience. Special consideration should be given to treatment vault design for MRgART machine, including, for example, RF shielding, quench pipe design, MR zoning, etc. all of which add extra cost in addition to regular radiotherapy machine shielding. The treatment unit itself is also expensive, higher than the cost of an MRI scanner and a linac combined.

Second, the MRgART introduces new risks due to MR hazards and uncertainties due to MR imaging used, thus demands re‐consideration in treatment strategies and even requires re‐designing of regular clinical workflow. Special staff training is required in order to understand MR hazards and emergency procedures with magnetic field presents. Patient MR screening now is mandatory and patient status needs to be closely monitored/rescreened for each fraction. Radiotherapy planning should take into account uncertainties and complexity resulting from the use of MR imaging, including imaging distortion, potential MR‐CT multi‐modality imaging registration errors, electron return effects, electron density mapping uncertainties, as well as dose calculation accuracy due to presence of MR field. Consequently, regular planning and delivery workflow might need to be redesigned to accommodate these concerns.

Moreover, long treatment and potential MR hazards’ limit the patient throughput for MRgART machines. It has been reported that a long treatment time slot is required for MRgART.^14^, ^15^ Due to MR safety concerns, some patients, for example, patients with pacemaker or ferromagnetic implants, cannot receive treatment on MRgART machines. All the above‐mentioned risks and uncertainties require extra precautions or staff training (or hiring MR physicists or MR technologists) for the treatment team which might not be possible at all for centers with limited resources, or small size centers that rely heavily on high patient volumes.

CT imaging, on the other hand, is a widely applied technology in radiation oncology practice and has been routinely used for many disease sites. Recent development of CTgART enables online adaptation with high‐quality FBCT or CBCT. CTgART offers several distinct benefits over MRgART. The above‐mentioned MR‐specific cost and concerns are largely eliminated. The integrated artificial intelligence (AI)‐assisted automatic segmentation and treatment planning process have greatly streamlined the ART workflow and improved overall treatment efficiency. Reasonable adaptive treatment time has been reported for the Varian Ethos platform, which allows potential high patient throughput with ART.^16^ One concern over CTgART is the low‐imaging quality for online delineation and the lack of soft‐tissue contrast for target visualization. Both FBCT and CBCT on the current ART platforms provide comparable image quality to the planning CT, which is already sufficient for many disease sites (head and neck, thorax, pelvis, spine, et al).^16^, ^17^ Admittedly, MR images still provide higher tissue contrast, yet MRgART is probably only essential for sites that can benefit from ART and require superior soft‐tissue contrast, for example, abdominal regions.

To conclude, as more centers adopt this less complex and more cost‐effective online adaption platform, the future mainstream for online adaptive radiotherapy will be using on‐board CT rather than on‐board MR images.

## 3 REBUTTAL

### 3.1 Daniel Hyer, PhD

Dr. Cai has argued against the future of MRI‐guided adaptive radiotherapy based on three main points: high capital cost, complex workflows, and low patient throughput. These arguments can be associated with nearly any new technology and are relevant for the present but not the future. I will counter each of these points in my rebuttal.

The capital costs associated with an MRI‐guided adaptive radiotherapy program include both construction and equipment costs. With respect to construction, it has been shown many times, including at our own center, that an MR‐linac can fit within the footprint of a standard linac vault. Some centers may require substantial work to receive these state‐of‐the‐art machines; but, from a historical perspective, these are one‐time costs that traditional radiotherapy departments will need to address in order to modernize. New radiotherapy departments will be constructed in a way to anticipate and accommodate MR‐linacs. While I will concede that the equipment costs will always be greater for a combined MRI and linear accelerator, this is only one part of the cost equation. In order to provide commensurate care achievable on an MR‐linac, clinics with only (CB) CT‐equipped devices will need to insert fiducials, beacons, spacers, etc. to address inadequacies in soft‐tissue imaging. The incremental costs associated with these devices will arguably exceed the upfront capital expenditures of an MR‐linac when summed over the decade or more in which they are used.

Dr. Cai's second argument is that MRI‐based workflows are more complex. I will agree that clinical workflows for adaptive radiotherapy are certainly more complex than an image‐guided radiotherapy (IGRT) workflow, but I do not believe that the choice of imaging modality makes a significant impact on the complexity of the workflow. The specific complexities that Dr. Cai mentions have all been addressed with modern MR‐linacs. Specifically, dose calculation algorithms used in MR workflows are based on well benchmarked Monte Carlo models which alleviate dosimetric concerns and image distortion is less than 1.1 mm in a 40 cm diameter spherical volume emanating from isocenter on the Elekta Unity.^5^ Dr. Cai also argues that registration in‐accuracies are unique to an MRI workflow; however, I will counter that registration in‐accuracies exist in the CT workflow due to the fact that MR images are commonly registered for tumor delineation in CT‐based planning. In cases where MR fusion is not performed, a clinic may be trading small registration uncertainties for larger targeting uncertainties.

The final point that Dr. Cai makes is that the throughput of MR adaptive radiotherapy is lower than that of CT adaptive radiotherapy. While I agree that in its current form, adaptive radiotherapy is less efficient than traditional IGRT approaches, I do not believe the selection of imaging modality makes a significant impact on throughput. Given the same software tools for registration, contouring, and treatment planning, the only time difference between CT and MR‐based adaptive radiotherapy is in the image acquisition. A standard MR image acquisition for a pelvis treatment at our site is 2 minutes. A similar pelvis CBCT acquisition can be up to 1‐minute in length, meaning that the difference between imaging time with on‐board MRI and on‐board (CB) CT treatments is on the order of 1 minute. When considering the entire treatment, the imaging time is only a small fraction of the entire process. Furthermore, it is reasonable to expect that segmentation, which is one of the most time‐consuming steps in the online adaptive process, will be more fully automated in the future on MR‐linacs because of the superior image quality.

In conclusion, my colleague argues that the strength of a CT‐guided approach is in its simplicity and low cost. This, however, is a view of the present challenges of MR guidance. Armed with a comprehensive set of MR sequences and techniques, I am confident that for many of our most challenging clinical problems, we will develop simpler and more cost‐effective approaches using the MR toolset than using the single imaging modality of (CB) CT. I look forward to a future where MR is the mainstay for adaptive radiotherapy.

### 3.2 Bin Cai, PhD

I appreciate Dr. Hyer's effort to introduce and discuss several advantages of MR imaging and its application in MRgART. I do agree that, as mentioned in my opening statement, MR images provide superior soft‐tissue contrast which is critical for accurate delineation of target and OARs of some treatment sites, that is, abdomen. I also agree it is hard to believe a single tool set or ART modality can resolve all challenging scenarios for every treatment site, therefore, in my opinion, both MRgART and CTgART will remain as powerful treatment approaches when implementing online ART. However, the debate topic here is which modality will be the future mainstream ART platform. I believe to serve as a mainstream platform, it should provide effective, efficient, and robust ART solutions for the majority of treatment sites, and also be easy to adopt by a wide range of clinical settings, from large academic cancer centers with great resources to small or standalone clinics with limited resources. As described previously, the MRgART poses more challenges in clinical implementation, which inevitably limits its wide adoptions particularly for centers with limited resources but large patient volume.

Indeed, superior MR imaging sequences can be deployed on MR‐Linacs for clear soft‐tissue visualization. However, the distinct benefits of these advanced imaging tools might be limited or specific to certain anatomical sites.^18^ Early adopters have shown the feasibility of CTgART for a variety of treatment sites where daily or periodic adaptation is needed, for example, HN, Pelvis, etc. Particularly, thanks to fast gantry speed, enhanced noise‐canceling grid, and advanced image reconstruction algorithm, much improved imaging quality of the latest CBCT has been observed even for upper GI areas.^19^, ^20^ Furthermore, “time” is a major limiting factor for online ART and each ART step needs to be performed in a timely manner to minimize the total treatment time, thus minimize the chances of anatomy change during the process. However, advanced MR imaging sequence often requires long imaging acquisition and reconstruction time, therefore, might not be appropriate for online ART. The concept of quantitative imaging with MRI is also exciting but this can be done with offline MRI which might not fit the pressing timeline during an online ART workflow.

From a technical point of view, Dr. Hyers mentioned several limitations on MR imaging: for example, image distortion, registration errors, and uncertainties in electron density mapping, and briefly argued that all limitations have been addressed. It is true that many research efforts have been put into these areas and potential solutions have been proposed. However, none of these solutions completely eliminate the uncertainties or errors for every treatment site. For example, the MR‐only workflow is promising but it is still new to the community. It definitely requires extra efforts in terms of validation and quality assurance. Many early adopters utilize their in‐house solutions on synthetic CT generation which requires high proficiency in programming; several commercial solutions are now available but often remain as a black box to the treatment team, therefore, require extra validation effort and extra precaution when used in a clinic.

Note that both commercial and research development of CT or CBCT technologies for the CTgART strategy have NOT reached their maturity yet. With new breakthroughs on X‐ray tube and detector design, improvement in imaging reconstruction algorithm as well as utilization of AI tools, there is no doubt that the image quality and integrity of (CB) CT imaging modality will be further improved. In addition, CT/CBCT imaging with contrast is still an option. As the entire field is going through this paradigm shift to hypo‐fractionation and ultra‐ hypofractionation, it is feasible to give contrast for a limited number of fractions during image acquisition for on‐board CT or CBCT to aid target delineation. Contrast CT or CBCT images will further extend the clinical utility of CTgART to cover more treatment sites, even those demanding superior soft‐tissue contrast.

CONFLICT OF INTEREST.

Daniel Hyer is the beneficiary of intellectual property licensed to Ion Beam Applications and provides consulting services for Elekta.
